# Beyond β-Adrenergic Receptor Brake: Compartment-Selective GRK2 Programs from Heart Failure to Cardio-Oncology

**DOI:** 10.3390/kinasesphosphatases4030019

**Published:** 2026-07-31

**Authors:** C. Reid Dotson, Lilly Underwood, Priscila Y. Sato

**Affiliations:** 1 Division of Cardiovascular Disease, Department of Medicine, Heersink School of Medicine, University of Alabama at Birmingham, Birmingham, AL 35233, USA

**Keywords:** GRK2, heart failure, β-adrenergic receptors, ischemia/reperfusion, cardio-oncology, anthracyclines, immune checkpoint inhibitors

## Abstract

Sustained neurohormonal stress, adverse myocardial remodeling, and inflammation are major components of heart failure (HF) progression. Alterations in circulating neurohormonal signaling are directly sensed by the β-adrenergic receptor (βAR), a system mainly responsible for chronotropic and inotropic cardiac responses. βARs are regulated by G-protein-coupled receptor (GPCR) kinase 2 (GRK2). Within this context, decades of study have unraveled mechanistic details on how GRK2 canonically imposes a “brake” on βARs and other GPCR-mediated signaling. Notably, an expanding body of evidence demonstrates that GRK2 functions in a highly compartment- and cell-dependent manner, with roles extending far beyond GPCR regulation. These noncanonical activities span metabolic control, maintenance of organelle integrity, and regulation of inter-cellular signaling networks, particularly those governing immune–vascular interactions. In this review, we will discuss recent advances in our understanding of the cell-specific functions of GRK2, its emerging biological roles in cardiac diseases, and the opportunities these findings present for advancing mechanistic insights in cardio-oncology. We propose that the therapeutic value of GRK2 is directly dependent on a deeper understanding of its noncanonical functions in a compartment- and cell-specific manner within a disease-specific context.

## 1. Introduction

Heart failure (HF) is a progressive cardiovascular disorder in which impaired cardiac function limits the heart’s ability to maintain sufficient blood flow to peripheral tissues. Paradoxically, chronic sympathetic nervous system (SNS) stimulation is a major driver of this process. While SNS activation supports early increases in cardiac output, chronic adrenergic signaling accelerates remodeling, promotes arrhythmias, and increases mortality ^1–3^. On the organ level, this causes the heart to gradually lose its ability to increase contractile strength. At the receptor level, cardiomyocytes undergo downregulation and uncoupling of βARs, limiting stress-induced increases in contractility, commonly described as loss of inotropic reserve ^2,4,5^.

GRKs directly regulate βAR desensitization through agonist-dependent receptor phosphorylation, which promotes β-arrestin recruitment and uncoupling from G-protein signaling ^6,7^. There are seven GRK isoforms, whereas the major isoforms in the heart are GRK2 and GRK5. For a detailed biochemical introduction of the GRK family and role of GRKs in GPCR-signaling, refer to this comprehensive review ^7^. Early studies in human HF showed that GRK2 expression and activity were increased in failing ventricles and that this increase coincided with impaired βAR responsiveness ^3,5^. Later work showed that GRK2 levels were elevated in peripheral blood cells, mirroring those in the myocardium and correlating with HF severity ^5,8^. These findings placed GRK2 as the nexus of sustained sympathetic drive and diminished adrenergic reserve in HF.

Importantly, recent studies have shown that GRK2 has a broader cellular impact that goes beyond the canonical “βAR brake.” These noncanonical functions of GRK2, including its role in metabolic remodeling, mitochondrial injury, immune–vascular signaling, and treatment-related cardiotoxicity, are discussed in later sections of this review. Table 1 summarizes localization, cell-type expression, and major contexts related to GRK2 addressed in this review.

## 2. GRK2—The “Brake” on βAR Signaling in Heart Failure

### 2.1. GRK2 Upregulation and Impaired βAR Responsiveness

GRK2 is a negative regulator of βAR and a major contributor to receptor desensitization in HF ^7^. Multiple studies have shown that in human HF, β1AR is diminished, with a concomitant decrease in responsiveness to β-agonism, a phenomenon linked to upregulation of GRK2 expression and activity ^1–3^. Using failing human myocardial tissue, an inverse correlation was observed between GRK activity and βAR-mediated cAMP generation ^5^. This was further supported by studies in mice overexpressing GRK2, in which βAR responsiveness was decreased ^14^. Thus, GRK2 was positioned as a contributor to βAR desensitization and the progressive loss of inotropic reserve in HF.

GRK2 is ubiquitously expressed, and its role as a receptor “brake” is essential to cardiac biology. In human HF, GRK2 expression and activity in circulating lymphocytes mirror those in the myocardium and are associated with reduced βAR responsiveness and HF severity ^5,6,8,13^. Thus, sympathetic overdrive influences GRK2 upregulation also in non-cardiac cells, including lymphocytes, which have been postulated as a biomarker for HF progression ^5,6,24–26^.

### 2.2. Chronic SNS Signaling, GRK2 Activation, and a Self-Perpetuating Cycle

Chronic SNS activation is a hallmark of HF and contributes to adverse outcomes ^4,24^. Sustained catecholamine exposure contributes to βAR downregulation and desensitization, with GRK2 activity contributing to this detrimental feedback loop. This increase in GRK2 activity leads to diminished βAR sensitivity and coupling, thereby decreasing responsiveness to βAR stimulation and subsequently compromising contractility. Decreased adrenergic response augments SNS activation, leading to further increases in circulating catecholamines and perpetuating signaling that promotes HF progression ^6,27^.

Thus, myocardial GRK2 levels correlate with clinical severity and cardiac functional readouts and are higher under conditions of augmented adrenergic stimulation, whereas decreased βAR signaling is associated with reduced GRK2 expression ^4,7,8^. Collectively, these findings support the concept that sustained sympathetic stimulation contributes to GRK2 upregulation and progressive impairment of βAR signaling in HF.

### 2.3. Reversal Logic: GRK2 Inhibition Restores βAR Signaling and Contractility

A major strength of the GRK2 “brake” model is its reversibility, that is, inhibiting GRK2 can partially restore cellular function. In failing cardiomyocytes, expression of βAR carboxy-terminus (βARKct) peptides, which act as competitive inhibitors to GRKs by binding Gβγ subunits, improves basal and β-agonist-stimulated cAMP production, restoring contractile responsiveness ^14,28^. This “reversal logic” is supported by multiple in vivo genetic and preclinical translational approaches ^7,29–32^. Cardiac expression of βARKct peptides enhances contractility and protects against adrenergic dysfunction in transgenic models and via viral-mediated gene delivery, establishing early proof of concept for GRK2 inhibition ^33,34^. Complementary gene-targeted studies showed that βARKct expression can prevent or delay the development of cardiac dysfunction in a genetic HF model, further supporting the potential of GRK2 inhibition as a therapeutic strategy ^14,29^.

In separate studies, by using a transgenic mouse model containing GRKInh, a peptide inhibitor of GRK2, Quitterer and colleagues showed that GRKInh mice exhibit cardioprotective effects post-myocardial infarction (MI) with reduced cardiomyocyte death and attenuated maladaptive metabolic remodeling ^30,35^. The serotonin-reuptake inhibitor (SSRI) paroxetine has been shown to inhibit GRK2 ^36,37^. Use of paroxetine in mice post-MI improves adrenergic contractile reserve and ventricular function ^32,36^. Moreover, an inhibitor derived from the paroxetine scaffold with higher selectivity for GRK2 produced enhanced dobutamine inotropic responses ^31^. These studies establish GRK2 as a central mediator of βAR dysfunction in HF and identify it as a therapeutic target to improve cardiac function ^3,28,30,32–37^.

## 3. GRK2 in Chronic HF: New Metabolic and Neurohormonal Roles

### 3.1. GRK2 as a Therapeutic Target in HF

HF treatment strategies have improved patient quality of life and mortality rates. Yet, these strategies delay disease progression without providing a cure. In fact, heart disease remains the number one cause of death in the US and the world. A large body of evidence demonstrates that GRK2 is consistently upregulated and activated across multiple HF models, and that its activity can de selectively modulated through interference of protein–protein interactions or inhibition of its catalytic function ^7,38^. As a result, several therapeutic strategies targeting GRK2 have been developed, including peptide-based inhibitors (i.e., βARKct, GRK2Inh), small-molecule approaches with GRK2-inhibitory activity (e.g., paroxetine), and strategies designed to prevent GRK2 recruitment (e.g., disruption of the Gβγ–GRK2 interface) ^28,32,37,39^.

While small-animal studies are important models that enable focused testing, preclinical experimentation is imperative for informing clinical trials. In a post-MI porcine model, AAV6 βARKct gene therapy supported GRK2 inhibitory strategies, improving left ventricular hemodynamics, and normalizing neurohormonal markers ^33^. Similar results were obtained in a mini-swine HF model using a novel GRK2 inhibitor ^31^. These findings position myocardial GRK2 inhibition as a viable long-term therapeutic strategy rather than merely a transient experimental intervention.

### 3.2. GRK2 and Cardiac Metabolism: Insulin Signaling and Glucose Utilization

HF is characterized by impaired myocardial energetics that lead to contractile dysfunction ^40^. GRK2 participates in linking neurohormonal stress and metabolic remodeling by inhibiting glucose uptake and promoting insulin resistance in the injured heart ^7,11,41,42^. This occurs in part through GRK2-mediated phosphorylation of IRS1, thereby limiting GLUT4 translocation and reducing glucose utilization ^41^. GRK2 can also translocate to mitochondria, where it regulates glucose oxidation ^12^. Thus, GRK2 integrates adrenergic signaling and metabolic regulation, highlighting its multifaceted role in HF progression.

GRK2-dependent cardiac metabolic dysfunction can also be therapeutically targeted. Pharmacologic and peptide-based GRK2 inhibition improves metabolic features and cardiac function in HF models, including improved markers of mitochondrial organization and contractile responsiveness ^43^. These studies provide a broader view of GRK2 as a remodeling kinase involved in physiological and pathological processes with a footprint that extends beyond βAR uncoupling to encompass the metabolic machinery that sustains myocardial performance during chronic disease ^12,41–43^.

More recently, studies on partial GRK2-biased β_2_AR agonists identified compound 15 to selectively enhance skeletal muscle glucose uptake via mTOR while minimizing Gs/cAMP signaling and β-arrestin recruitment ^44^. This compound improved glucose homeostasis as measured by glucose tolerance tests. It also improved fat and lean mass with minimal cardiac atrial contractility impact when combined with GLP-1 therapy ^44^. Notably, long-term in vivo global metabolic impact of compound 15 remains to be fully characterized. This is particularly relevant as β-cell-specific GRK2 knockout increase insulin sensitivity as measured by hyperinsulinemic-euglycemic clamps ^42^, global-GRK2 heterozygous mice have decreased weight gain in an obesogenic regimen and increased insulin sensitivity ^45^, and GRK2 inhibition has been proposed to potentiate GLP1-receptor agonism ^46^. Overall, the studies support an important role for GRK2 in metabolic regulation.

### 3.3. Adrenal GRK2 and Systemic Sympathetic Overactivity

Adrenal regulation is a major determinant of circulating catecholamine levels, and dysregulation of this organ amplifies systemic adrenergic stress. Mechanistic studies show that upregulation of adrenal GRK2 desensitizes α2AR in chromaffin cells, thereby increasing catecholamine release and worsening sympathetic overdrive ^24^.

Adrenal-targeted GRK2 inhibition or gene deletion reduces circulating catecholamines, improving post-MI cardiac βAR signaling and function ^25,47^. Moreover, exercise training, an established HF treatment, has also been linked to restoration of the adrenal GRK2/α2AR axis, offering an additional benefit in reduced sympathetic overdrive in trained HF models ^26^. Together, these findings support a multi-compartment view of GRK2 biology in HF, in which myocardial and adrenal GRK2 influence both local signaling and systemic neurohormonal activation.

## 4. Ischemia/Reperfusion (I/R): Compartment-Specific GRK2 Signaling, Cell Death, and Metabolic Outcomes.

### 4.1. Acute I/R: Signs of GRK2 Degradation and Collapse of Pro-Survival Signaling

**Figure 1.**
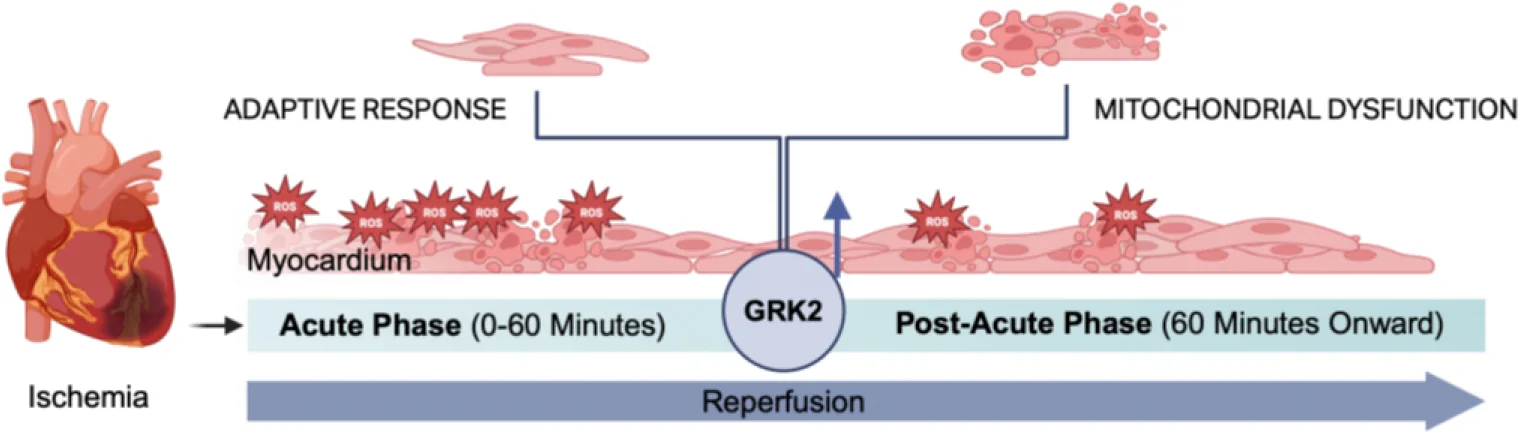
Timeline representation of acute and post-acute I/R myocardial events linked to GRK2. Created in BioRender. Dotson, R. (2026) https://BioRender.com/0rziyfg.

I/R injury involves many signaling pathways that are major determinants of cellular outcomes including activation of kinase cascades and proteostasis programs. The acute phase of I/R injury spans from minutes up to 1 h and is reported to involve phosphorylation-dependent GRK2 degradation, coupled with concurrent loss of pro-survival Akt signaling and Pin1 ^9^. This pattern highlights that even during the acute stages of reperfusion, there are subtle but important changes in signaling homeostasis that impact cell fate, reinforcing that “timing” is central for intervention during the reperfusion phase of I/R injury. Figure 1 illustrates acute phase changes pertinent to I/R.

### 4.2. Post-Acute I/R: ERK-Dependent Ser670 Phosphorylation, Hsp90 Binding, and Mitochondrial Targeting

During the post-acute stages of I/R (post-60 min), ERK-dependent phosphorylation of GRK2 at Serine 670 promotes Hsp90 binding and mitochondrial translocation, thereby promoting cardiomyocyte death ^11,48^. Notably, the association of Hsp90 with GRK2 is a key determinant for the proper maturation and stability of the kinase ^48^. Thus, localization, abundance, and phosphorylation status of GRK2 are dynamically altered during the reperfusion phase and contribute to I/R injury. Figure 1 illustrates post-acute changes pertinent to this reperfusion phase.

### 4.3. Limiting Mitochondrial GRK2 Preserves Glucose Oxidation and Reduces Injury

Genetic models provide compelling evidence that mitochondrial translocation of GRK2 contributes to post-I/R dysfunction. Mice harboring the GRK2 Ser670Ala (S670A) mutation, which hinders phosphorylation-dependent mitochondrial targeting of GRK2, exhibit reduced cardiomyocyte death and improved cardiac functional recovery post 24h of IR ^12^. This effect is partly attributable to the prevention of GRK2 mitochondrial translocation, which preserves glucose oxidation and maintains pyruvate dehydrogenase activity ^12^. Importantly, because this mutation is expressed ubiquitously, potential effects in non-cardiac tissues and their indirect contributions to the observed cardiac phenotype must also be considered. Nonetheless, data support that mitochondrial GRK2 is an important contributor to post-ischemic metabolic dysfunction.

### 4.4. Multi-Cellular Regulation of I/R: Cardiomyocyte vs Fibroblast GRK2

I/R outcomes depend on coordinated responses across multiple cardiac cell types. GRK2 directly influences cardiomyocyte cell survival, as cardiomyocyte-specific genetic ablation of GRK2 reduces apoptosis, limits infarct size, and improves functional recovery following I/R ^49,50^. Additionally, GRK2 in cardiomyocytes is involved in mitochondrial cell death pathways ^9,11^. GRK2 in cardiac fibroblasts regulates acute immune-cell recruitment and promotes subsequent remodeling responses. Fibroblast-specific GRK2 knockout reduces infarct size, preserves cardiac function, decreases fibrosis, and is associated with reduced neutrophil infiltration and TNF-α signaling ^10^. These observations suggest that GRK2 functions across multiple cardiac cell types to regulate injury-induced immune responses and repair mechanisms, shaping both the severity of the initial insult and the long-term remodeling outcome ^10,49^.

### 4.5. NO/S-Nitrosylation Cross-Talk Converges on GRK2

Nitric oxide (NO) is a critical mediator of cardioprotection during I/R injury. While during reperfusion excessive ROS generation contributes to cellular damage, physiological NO signaling can limit injury by improving coronary perfusion, reducing mitochondrial dysfunction, and attenuating inflammatory responses ^51,52^. One important mechanism underlying these NO protective effects is protein S-nitrosylation, a reversible post-translational modification in which NO covalently modifies cysteine residues, thereby altering protein activity, localization, and protein–protein interactions ^53^. GRK2 is regulated by redox-dependent modifications. In particular, S-nitrosylation of GRK2 at cysteine 340 (C340) inhibits its kinase activity, preventing excessive receptor desensitization and maladaptive signaling during cardiac stress ^54,55^. During I/R injury, NO-mediated nitrosylation of GRK2 C340 has been proposed as an endogenous cardioprotective mechanism that limits GRK2 activation, preserves β-adrenergic signaling, and reduces myocardial injury ^54,55^. Loss of this regulatory modification exacerbates cardiac dysfunction, highlighting the importance of GRK2 C340 nitrosylation as a key node linking NO signaling to cardioprotection ^54,55^. Notably, this NO-modification in GRK2 is also linked to age-related cardiac dysfunction ^56^.

## 5. Immunovascular GRK2 Programs: Endothelium, Leukocyte Trafficking, and Macrophage Immunometabolism

### 5.1. Endothelial GRK2: Adhesion Programs and Inflammatory Recruitment

Cardiac injury and remodeling are modulated by cardiomyocyte stress responses and by immune-cell recruitment. The vascular endothelium is a key gatekeeper in this process, regulating mechanical forces and inflammatory cues that control leukocyte adhesion and migration. Recent mechanistic work shows that disrupted blood flow promotes GRK2 Serine 29 phosphorylation with downstream AP1 activation, increasing ICAM 1/VCAM 1 expression while decreasing AMPK signaling, linking endothelial GRK2 to both inflammatory adhesion programs and metabolic tone ^15,16^. Serine 29 of GRK2 is located within the calmodulin binding domain, and phosphorylation by PKC has been shown to abolish GRK2 inhibition by calmodulin ^57^. Causal support for an endothelial role of GRK2 also comes from knockout mice with endothelium-selective knockout of GRK2 ^58^. Endothelial GRK2 deficiency impaired vascular homeostasis, enhanced mitochondrial ROS production and inflammatory signaling, and led to structural and functional vascular dysfunction ^58^.

### 5.2. GRK2 and the Akt/eNOS Axis

In parallel with adhesion signaling, endothelial homeostasis is critically governed by NO production. Mechanistic evidence links GRK2 to endothelial NO signaling by interactions with Akt, affecting eNOS activation and NO bioavailability ^54,59^. This provides a molecular basis for shared NO-GRK2 signaling axis across vascular and myocardial compartments ^54,59^.

### 5.3. Leukocyte Migration: GRK Networks and Chemokine GPCR Regulation

**Figure 2.**
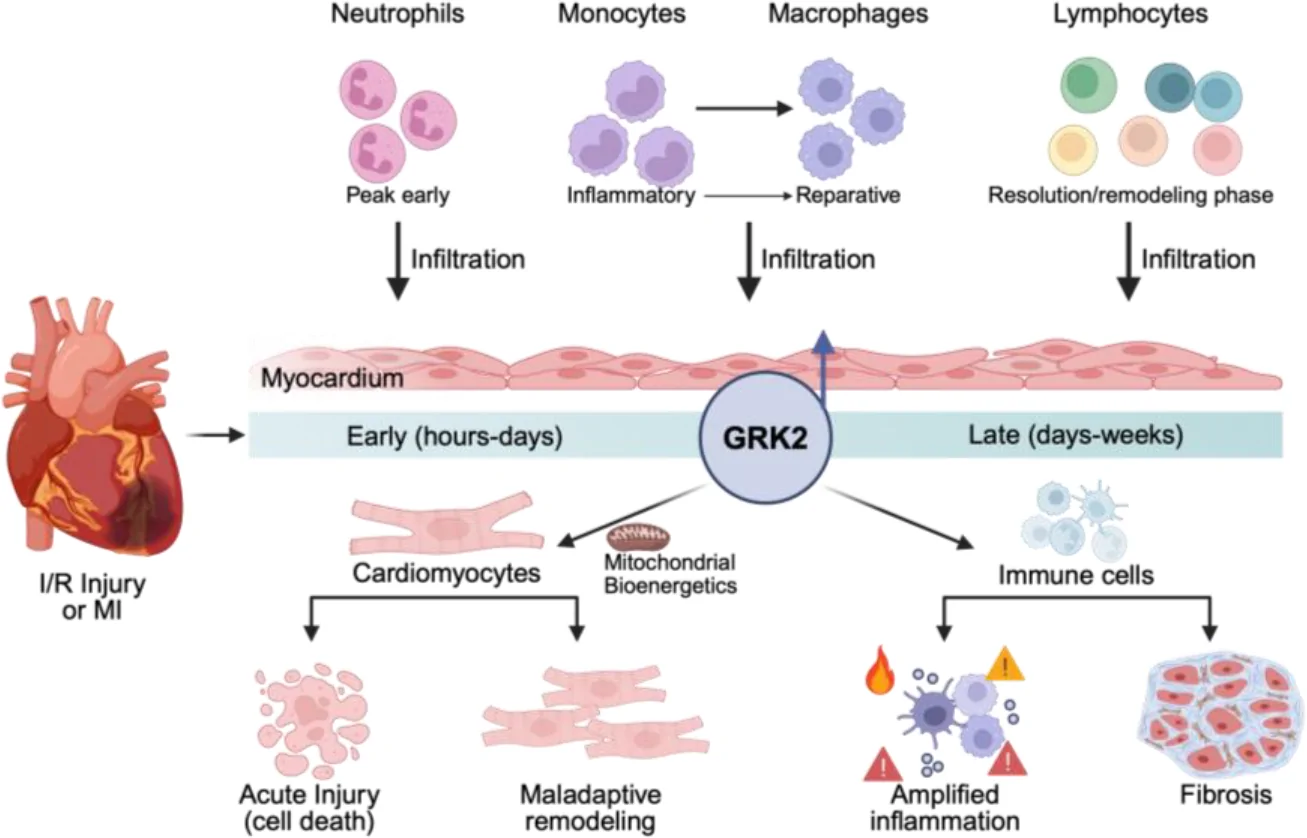
Schematic illustration of early and late immune events in response to MI. Created in BioRender. Dotson, R. (2026) https://BioRender.com/fws6w8l.

The healthy myocardium contains resident macrophage populations that persist following injury and shape the early inflammatory environment ^60^. After MI, neutrophils are among the first blood-derived leukocytes to enter the myocardium. Resident cardiac macrophages help drive this early extravasation through TLR9/MyD88/CXCL5 signaling ^61^. Monocyte recruitment occurs in two phases, with Ly6C^hi^ monocytes dominating the early inflammatory phase and Ly6C^lo^ monocytes accumulating later to support angiogenesis and collagen-based repair ^62^. Studies in rheumatoid arthritis, Weibel-Palade body formation, and multiple sclerosis have suggested that GRK2 protein levels in leukocytes may control the intensity and duration of certain chemokine-triggered signaling ^17,63–65^. An illustration of timing and immune contribution post-MI is shown in Figure 2.

Once leukocytes enter the tissue, their migration and positioning depend on chemokine GPCR signaling and receptor desensitization, both of which are regulated by GRKs. Combinatorial GRK depletion studies showed that leukocyte migration depended on specific GRK isoform involvement, emphasizing that GRK2 operates within an extensive GRK network that tunes immune-cell trafficking in a context- and cell-type-dependent manner ^19^. Nonetheless, the specific role of GRK2 in leukocytes in the context of MI remains less understood and warrants further investigation.

### 5.4. GRK2 Framework in the Context of Macrophage Immunometabolism

After the initial neutrophil wave, circulating monocytes are recruited to the injured myocardium via chemokine signaling. Upon entering the tissue, monocytes differentiate into a spectrum of macrophage states with distinct functional properties ^66^. In murine MI, Ly6C^hi^ monocytes dominate the early inflammatory phase (roughly days 1–4), clearing necrotic debris and modulating proteolytic and inflammatory signaling (Figure 2). As inflammation resolves, Ly6C^lo^ monocytes and macrophages become more prominent and support efferocytosis, angiogenesis, myofibroblast expansion, and collagen deposition ^62,67^.

Macrophage behavior depends on both lineage and the local metabolic milieu. Their activation states are tightly linked to metabolic reprogramming; a process that may be linked to GRK2 biology. Glycolysis, oxidative phosphorylation, and related metabolic pathways help distinguish inflammatory from reparative macrophage programs ^68,69^. GRK2 has been linked to pathways that influence macrophage polarization and glycolysis, including EP4-cAMP-pCREB signaling and PKM2-related metabolic regulation ^17,18^. These findings raise the possibility that GRK2 may influence macrophage behavior during tissue injury.

### 5.5. Adaptive Immune Recruitment: Delayed Lymphocyte Accumulation and Potential GRK2 Control Points

Adaptive immune-cell recruitment follows the early innate immune response and becomes increasingly evident during the first week post-MI (Figure 2). Flow cytometric analyses in murine MI models show that CD8^+^ T-cells, γδ T-cells, B cells, and myeloid dendritic cells peak around day 7 post-MI ^70^. In the same setting, Th1 cells and regulatory T-cells (Tregs) are the predominant CD4^+^ T-cell subsets in the infarcted myocardium ^70^. Timely reperfusion attenuates this later adaptive immune wave, supporting the idea that lymphocyte recruitment is temporally downstream of the initial innate response.

Recent transcriptomic analyses refine this view by showing that the healthy heart already contains resident or tissue-associated B- and T-cell populations and that, following MI, these cells sustain inflammatory and remodeling-associated programs ^71^. Post-MI, cardiac T-cells upregulate transcripts associated with cytokine production and cytotoxicity, including Ccl5, Ifng, Tnf, Gzmk, and Prf1, whereas B cells upregulate activation-associated and matrix-related programs, including Il6, Il1rn, Ccl6, and collagen transcripts ^71^. Notably, these altered transcriptional states can last beyond the initial lymphocyte wave (Figure 2; days 5–7), supporting a role for the adaptive arm in the transition from early injury to chronic remodeling.

Although the role of GRK2 in cardiac lymphocytes following ischemic injury remains poorly defined, existing evidence points to several mechanisms through which GRK2 may influence adaptive immune responses in the injured myocardium. In primary immune cells, combinatorial GRK depletion studies show that GRK2, together with other GRKs such as GRK6, regulates chemokine receptor signaling in a cell-type- and receptor-dependent manner, altering both T-cell and B-cell chemotaxis and tissue localization in vivo ^19^. T- and B-cell infiltration into the myocardium depends on coordinated chemokine gradients and their cognate receptors, suggesting that GRK2-mediated receptor desensitization likely influences immune-cell recruitment and timing post-MI.

Beyond migration, GRK2 can also directly regulate T-cell effector signaling. In human T-cells, GRK2 is required for T-cell receptor (TCR)-induced transactivation of CXCR4, formation of a TCR-CXCR4 signaling complex, recruitment of PI3Kγ/PREX1, and robust cytokine secretion ^19,72^. Conversely, GRK2 depletion or pharmacologic inhibition attenuates these responses ^19,72^. Moreover, GRK2-mediated desensitization of the Sphingosine-1-Phosphate Receptor 1 (S1PR1) is critical to both T-cell maturation and blood chemotaxis ^73^. Together, these data support a working model in which GRK2 may influence not only the timing and extent of adaptive immune-cell entry into the injured heart but also the functional output of infiltrating T-cells once they populate the tissue. GRK2 in adaptive immune cells may integrate chemokine responsiveness, antigen receptor cross-talk, and cytokine output to shape the evolution from acute inflammation to persistent immunopathology.

## 6. Anthracycline Cardiotoxicity: Trial Evidence, Risk Stratification, and Mechanism-Linked Biomarker Opportunities

### 6.1. Clinical Framework and Definitions

Anthracyclines remain a cornerstone of cancer therapy, but their clinical use is constrained by cumulative myocardial injury that can progress from subclinical biomarker and imaging abnormalities to overt HF. In contemporary cardio-oncology management, this spectrum is commonly captured under the term cancer therapy-related cardiac dysfunction (CTRCD). Current guidance from the European Society of Cardiology (ESC) and the International Cardio-Oncology Society (IC-OS) has helped standardize definitions, severity grading, and surveillance pathways across studies and clinical practice ^74,75^.

The 2022 ESC cardio-oncology guidelines and the IC-OS consensus statement provide the current clinical system for assessing anthracycline-associated cardiovascular injury ^74,75^. These documents harmonize definitions of CTRCD, myocarditis, vascular toxicity, arrhythmias, and related complications, and recommend risk-adapted monitoring using cardiac biomarkers, echocardiography, global longitudinal strain (GLS), and selected use of cardiac magnetic resonance imaging. It is because of this precise monitoring structure that cardio-oncology cohorts provide the ideal platform to meaningfully assess GRK2’s diagnostic and prognostic value.

### 6.2. Troponin-Guided Strategies: Classic Evidence and Modern Boundary Conditions

Cardiac troponins are highly sensitive biomarkers of myocardial injury, reflecting the release of intra-cellular contractile proteins from cardiomyocytes into the circulation in response to cellular damage or stress ^76^. The development of biomarker-guided cardioprotection in cardio-oncology was driven by studies showing that troponin elevation identifies early myocardial injury. Cardinale and colleagues showed that patients with troponin I elevation after high-dose chemotherapy had a smaller decline in left ventricular ejection fraction (LVEF) when the ACE-inhibitor enalapril was started early ^77^. Although the study established the feasibility of injury-guided cardiac intervention, its findings cannot be readily generalized to routine prophylactic use in standard anthracycline-treated populations. The enrolled patients were identified by post-treatment troponin elevation after high-dose chemotherapy, a population distinct from those receiving conventional adjuvant anthracycline therapy.

Subsequent trials refined the limits of that approach in lower-risk and more contemporary anthracycline-treated populations. In Cardinale’s “ICOS-ONE” study, starting enalapril in all patients before chemotherapy was not superior to a troponin-triggered strategy in which enalapril was introduced after biomarker elevation was observed. This suggests that universal prophylaxis may not an added value in relatively low-risk patients receiving modest cumulative anthracycline doses ^78^. Likewise, in the “Cardiac CARE” study by Henriksen and colleagues, high-sensitivity cardiac troponin I was used to identify patients at higher risk during anthracycline therapy, but subsequent cardioprotection with carvedilol plus candesartan did not prevent short-term decline in LVEF ^79^. Together, these studies suggest that troponin remains highly valuable for risk enrichment and injury detection. However, translating troponin positivity into effective, uniform preventive pharmacotherapy has been more difficult than early proof-of-concept studies implied.

### 6.3. Neurohormonal Prophylaxis: Modest, Endpoint-Dependent Benefit

Despite being the more conventional approach, prophylactic neurohormonal blockade has yielded mixed results that vary according to the endpoint evaluated. In the Prevention of Cardiac Dysfunction During Adjuvant Breast Cancer Therapy (PRADA) trial, concomitant candesartan attenuated the early decline in LVEF during adjuvant anthracycline-containing therapy for early breast cancer, whereas metoprolol alone did not improve the primary imaging endpoint. Extended follow-up suggested some favorable effects of candesartan on ventricular remodeling and strain, but at 2 years, it had not prevented the decline in LVEF ^80^.

A similar pattern was observed in the Carvedilol for Prevention of Chemotherapy-Related Cardiotoxicity (CECCY) trial, which tested carvedilol in women with HER2-negative breast cancer receiving anthracycline-based chemotherapy. Carvedilol did not reduce the primary endpoint of early LVEF decline, but it was associated with lower troponin release and less diastolic dysfunction ^81^. Taken together, PRADA and CECCY suggest that neurohormonal prophylaxis may confer modest biological or subclinical benefit, but the magnitude and clinical relevance of that benefit depend heavily on the endpoint assessment, the baseline cardiovascular risk of the population, and the anthracycline regimen itself. This is likely why current practice favors selective use in higher-risk patients rather than a blanket prophylaxis for all.

### 6.4. Dexrazoxane: Most Consistently Supported Pharmacologic Prevention

Compared with other pharmacologic strategies, dexrazoxane has the most consistent evidence supporting its ability to prevent anthracycline cardiotoxicity ^82,83^. The mode of action for dexrazoxane is not entirely understood, but it is postulated that at least in part, it acts as an iron-chelator, involved in reducing ROS. Randomized trials in advanced or metastatic breast cancer showed that dexrazoxane reduced cardiac events and symptomatic HF. In those studies, there was no clear evidence that it reduced antitumor efficacy or tumor response ^83^. Recent systematic studies further support reduced HF and cardiotoxicity in adults receiving anthracycline-based therapy, with no clear evidence of diminished oncologic benefit ^84,85^.

The need for cardioprotective strategies is greatest when anthracycline therapy cannot be interrupted due to oncologic necessity. This is especially true for patients with high cumulative drug exposure or previous anthracycline treatment, which elevates the risk of cardiac dysfunction. Dexrazoxane stands apart from beta-blockers and renin–angiotensin system (RAS) inhibitors because it has the strongest direct evidence for reducing cardiac injury while allowing anthracycline treatment to continue. To date, there are no studies on dexrazoxane’s impact on GRK2 or vice versa. However, dexrazoxane’s proposed mechanism of action involves preventing the formation of oxygen-free radicals ^86^ and mitochondrial GRK2 upregulation promotes superoxide and ROS ^11,38^. Although we speculate a potential mechanistic convergence, the role of cardiac GRK2 in this context remains unknown but warrants further investigation.

### 6.5. Translational Niche: GRK2 as a Candidate Biomarker Axis in Cardio-Oncology

Anthracycline surveillance is valuable not only for guiding early preventive care but also for serving as a clinically well-defined platform for the validation of translational biomarkers. Patients are longitudinally monitored with troponins, natriuretic peptides, echocardiography, GLS, and, in some cases, cardiac magnetic resonance imaging ^79,80,87^. The longitudinal surveillance schedule provides an opportunity for repeated sampling time points at which GRK2 expression can be evaluated ^88^. By capturing compartment-specific stress responses, myocardial or immune-cell GRK2 may provide information not reflected by current surveillance biomarkers. Cardio-oncology cohorts offer a unique opportunity to test whether GRK2 improves the detection and risk stratification of cardiotoxicity, especially when traditional biomarkers are inconclusive.

## 7. Immune Checkpoint Inhibitor (ICI) Myocarditis: Spectrum, Diagnostics, Mechanisms, and Treatment Escalation

### 7.1. Clinical Phenotype and Registry Outcomes

ICIs are revolutionary immunotherapy drugs that are used to treat patients with a variety of cancers. Among the toxicities identified with ICI therapy, myocarditis has emerged as a clinically significant adverse event. Early reports indicate that this primarily arises from patients receiving combinatory immune checkpoint blockade ^89^. Multicenter registry data showed myocarditis often occurs early after initiation of therapy, can present without reduction in EF, and carries high rates of major adverse cardiac events (MACE), supporting the need for early recognition and management ^90^. Evidence from large clinical trial datasets estimates ~1% of patients had ICI-induced cardiovascular adverse events where myocarditis mortality was 38%, highlighting the necessity for standardized diagnostic and treatment methods ^87^.

### 7.2. Risk Factors and Thymic Predisposition

Among clinical risk factors, concurrent PD-1 and CTLA-4 blockade has been repeatedly associated with a heightened incidence of myocarditis ^90^. High-impact evidence points to thymic alterations as susceptibility factors, with thymic epithelial tumors associated with increased incidence/severity and earlier onset of overlapping “myotoxicity” syndromes ^91^.

### 7.3. Diagnostic Work-Up: Troponin, Imaging, and Endomyocardial Biopsy (EMB) Grading

Clinical presentation is highly variable, posing challenges for accurate and timely diagnosis. Although current diagnostic approaches rely on a combination of biomarkers and multimodal imaging, EMB remains the definitive diagnostic standard, especially in ambiguous cases. Histological evaluation of EMB specimens from a large ICI-treated cohort demonstrated a continuum of myocardial inflammation, indicating that some patients with low-grade disease may tolerate ongoing ICI therapy without immunosuppressive treatment and underscoring the value of risk-stratified management ^92^. Baseline troponin measurement before the initiation of ICI therapy, together with serial monitoring during treatment, is emerging as a practical strategy for the early detection of cardiovascular toxicity. Despite its potential utility, this strategy is not currently considered standard of care and requires additional validation before it can be integrated into routine clinical standard of care for myocarditis detection ^93^.

### 7.4. Mechanisms: Immune Tolerance, Macrophage Programs, and the IFNγ Axis

Direct evidence linking GRK2 to ICI myocarditis remains limited, yet current pathogenic models center on immune regulatory pathways in which GRK2 has been shown to play a role. This includes chemokine-dependent immune-cell trafficking, macrophage inflammatory programming, and amplification of cytokine receptor signaling ^18,19,72,73^. Mechanistic studies show that checkpoint pathways are vital for immune tolerance in cardiac tissue and that loss of inhibitory signaling can unleash autoreactive responses ^94^. Translational murine and human tissue studies support expansion of inflammatory macrophage subpopulations (CXCL9/10-high) induced by IFNγ signaling and highlight the interaction with CD8^+^ T-cells as critical to myocarditis pathogenesis. This identifies the IFNγ axis as a potential target in severe cases ^95^.

Recent work has identified a CXCL9/10^+^ CCR2^+^ macrophage–CXCR3^hi^ CD8^+^ T-cell axis in ICI myocarditis, supporting the concept that inflammatory cardiac macrophages help sustain cytotoxic T-cell recruitment and retention within the myocardium ^96^. In preclinical models, both depletion of these macrophages and pharmacologic CXCR3 blockade reduced myocardial T-cell infiltration and improved survival. Human cardiac biopsy samples showed similar enrichment of CXCR3^+^ T-cells and CXCL9/CXCL10^+^ macrophages ^96^. These studies support a contributing role in disease amplification and represent a potential therapeutic target. Collectively, these mechanistic observations suggest that ICI myocarditis arises within an immune signaling landscape that overlaps considerably with established GRK2 function. Notably, separate studies have shown that GRK2 regulate CXCR3 recruitment and modulation post-stimulation ^97^. Given the central roles of inflammatory macrophages and cytotoxic T-cells in disease pathogenesis, GRK2 may be an important regulator of immune-cell trafficking and activation within the inflamed myocardium.

### 7.5. Treatment: Steroids First Line; Escalation and Emerging Trials

First-line therapy for ICI-induced myocarditis remains high-dose corticosteroids; refractory cases may require additional immunosuppression. Case-level evidence supports abatacept (CTLA 4 agonism) for severe steroid-refractory myocarditis, and clinical updates emphasize ongoing trials evaluating abatacept and other steroid-sparing approaches ^98,99^. Notably, multicenter registry analyses suggest that both earlier initiation and higher initial steroid dosing improved outcomes with reduction in major adverse events ^100^.

## 8. Therapeutic Windows and Translational Design Principles: Compartment- and Cell-Selective GRK2 Targeting

### 8.1. Timing Matters: Acute vs Post-Acute Injury Signaling

**Figure 3.**
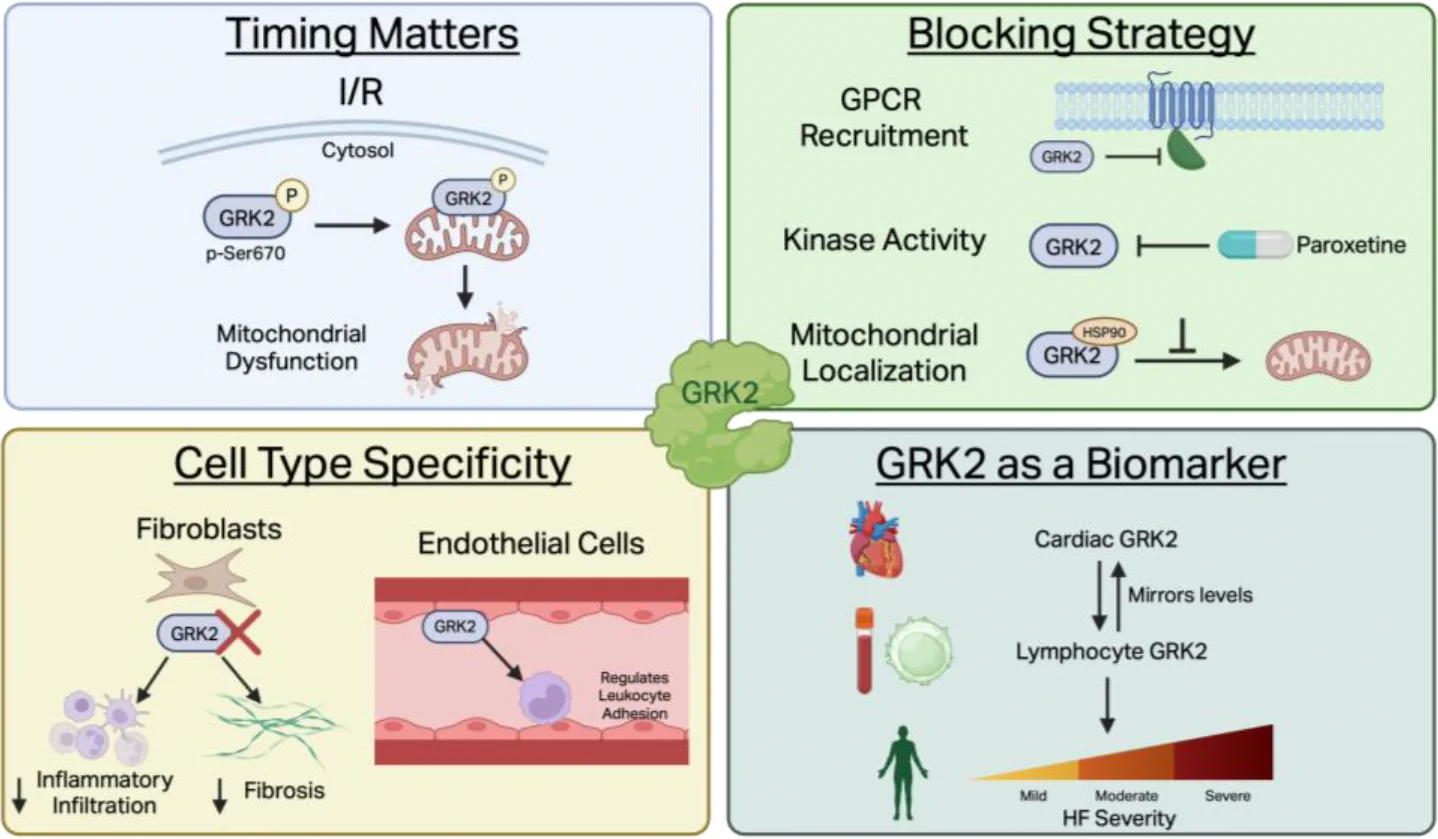
Schematic illustration for therapeutic windows where GRK2 pharmacological intervention may be advantageous in disease progression. Created in BioRender. Dotson, R. (2026) https://BioRender.com/7vf38uz.

I/R studies show that GRK2 cardiac signaling is not monolithic: initial acute injury features proteostasis-linked GRK2 degradation and loss of survival signaling, while post-acute injury involves serine 670 phosphorylation and mitochondrial trafficking that drives cell death and metabolic impairment (Figure 3) ^9,11,12^. This evidence suggests exploring phase-specific interventions to preserve protective signaling, block mitochondrial GRK2 localization, limit injury, and preserve cardiac function.

### 8.2. Compartment Logic: “Block Recruitment,” “Block Catalysis,” or “Block Trafficking”

GRK2 can be targeted at multiple mechanistic levels: preventing recruitment to activated GPCRs (βARKct; Gβγ interface inhibitors), inhibiting catalytic activity (small molecules such as paroxetine), or selectively blocking mitochondrial trafficking (Ser670/Hsp90 axis; Figure 3) ^11,39,101^. Each approach likely produces distinct physiologic and safety characteristics, and the most appropriate strategy may differ in chronic HF, acute I/R, and cardio-oncology cardiotoxicities.

### 8.3. Cell Specificity: Fibroblast and Endothelial Targeting as Remodeling Modifiers

Fibroblast GRK2 deletion reduces inflammatory infiltration and fibrosis post-I/R, while endothelial GRK2 regulates adhesion programs that govern leukocyte entry ^10,16^. This supports cell-type-directed delivery or cell-selective regulatory nodes for therapeutic development—particularly for inflammation-dominant remodeling phenotypes (Figure 3).

### 8.4. Biomarker Integration: Peripheral Blood Mononuclear Cells (PBMCs) GRK2 Within Guideline Surveillance Frameworks

Human HF data demonstrate that PBMC and lymphocyte GRK2 levels mirror myocardial levels and track severity, and that exercise-associated changes in lymphocyte GRK2 may have prognostic relevance ^5,13,88,102^. Translating this concept to cardio-oncology would require rigorous prospective validation alongside established clinical monitoring (troponin/BNP, echo/GLS, CMR in selected cases) as emphasized by current guidelines and consensus definitions ^74,75^ (Figure 3).

## 9. Conclusions and Future Directions

GRK2 is uniquely positioned at the intersection of adrenergic receptor regulation, myocardial metabolism, mitochondrial death signaling, and immunovascular biology. Human HF studies established GRK2 elevation as a correlate of adrenergic dysfunction and severity, while genetic and translational interventions demonstrate causality and therapeutic promise ^3,5,29,31,33^. I/R injury studies reveal compartment-specific mechanisms (proteostasis-linked early effects; Serine670/Hsp90 mitochondrial trafficking later) that highlight actionable levers ^9,11,12^. Multi-cellular studies in fibroblasts and endothelium point to system-level effects on immune recruitment and remodeling ^10,16^.

In cardio-oncology, anthracycline and ICI cardiotoxicities underscore the importance of risk stratification, biomarker interpretation, and targeted escalation strategies, while revealing that generalized prophylaxis often yields modest benefits and should be mechanistically and clinically enriched ^78,80,81,87,90,100^.

Several priorities should guide future GRK2-directed translational research. First, patient stratification will likely need to move beyond single biomarkers and instead incorporate multi-parameter frameworks that combine troponin kinetics, global longitudinal strain, clinical risk features, metabolic characteristics, and mechanistically informative markers of immune activation ^75,93^. Second, the development of compartment-selective GRK2 modulation remains an important goal, particularly strategies that limit pathologic mitochondrial GRK2 accumulation after injury without altering physiologic GPCR desensitization ^11,12^. Third, increasing evidence from endothelial and fibroblast studies suggests that cell-type-directed delivery may offer a way to modulate immune recruitment and fibrosis while limiting systemic effects ^10,16^. Finally, the potential of GRK2 as a biomarker axis should be tested prospectively. This includes PBMC GRK2 and GRK2-linked functional assays in both HF and cardio-oncology cohorts using guideline-aligned clinical and imaging endpoints ^5,13,75^. Beyond its established role in GPCR desensitization, GRK2 has emerged as a multifunctional signaling molecule whose noncanonical activities may contribute directly to disease pathogenesis, presenting new opportunities for therapeutic targeting in context-specific pathologies.

## Figures and Tables

**Table 1.** GRK2 role in context- and cellular-type-dependent compartmentalization (OSS = Oscillatory-Shear Stress; DOX = doxorubicin; ICI = immune checkpoint inhibitor).

Context	GRK2 Level/State	Compartment(s)	Immune Effects	Metabolic Effects	Biomarker Potential	Therapeutic Targets	Key Refs
**Acute I/R**	GRK2 degradation; Pin1/AKT loss	Membrane ↓; Mitochondrial trafficking risk	Neutrophil influx shaped by fibroblast GRK2	AKT pathway suppression	—	Preserve GRK2/AKT; inhibit calpain/proteasome (timed)	^9,10^
**Post-Acute I/R**	ERK → Ser670; Hsp90 → mito GRK2	Mitochondria ↑	Pro-death signaling; mPTP opening	ROS ↑; Apoptosis ↑	—	Block Ser670; disrupt Hsp90 docking; mPTP modulators	^11,12^
**Chronic HF**	GRK2 ↑ (myocardium, lymphocyte)	Membrane & Mitochondria	β-AR dysfunction; lymphocyte GRK2 tracks severity; exercise reduced	FA catabolism altered; β-AR-mitochondrial response impaired	PBMC GRK2 predicts survival	βARKct, paroxetine; gene therapy; exercise	^8,13,14^
**Endothelium (OSS)**	GRK2 S29 phosphorylation	Nucleus/AP-1 axis	ICAM1/VCAM1 ↑ → leukocyte adhesion	AMPK ↓ via NR4A1/LKB1	—	Target GRK2/AP-1; shear normalization	^15,16^
**Macrophage Polarization**	GRK2 translocation; EP4 desensitization	Membrane ↔ Cytosol	M2 suppressed when GRK2 ↑; paroxetine restores	PKM2 (S406/K433) → glycolysis ↓	—	GRK2 inhibitors; EP4 agonism; metabolic co-therapy	^17,18^
**Lymphocyte/ Neutrophil Migration**	GRK2 with GRK3/5/6 networks	Membrane	Chemotaxis tuning; cell-type specific	—	PBMC GRK2 (HF)	Combinatorial GRK targeting	^19,20^
**Anthracycline (DOX)**	Early GRK2 ↓ (heart, PBMC)	Membrane/Cytosol	Endothelial & immune changes precede damage	Mitochondrial stress; Ca^2+^ dysregulation	Early sentinel	Serial PBMC GRK2; cardio-onc surveillance	^21,22^
**Immunotherapy (ICI)**	Unknown baseline GRK2 signature	Immune Cells	Myocarditis/pericarditis via T-cell hyperactivation	—	GRK2 as risk stratify	Prospective biomarker + chemotaxis assays	^23^

## Data Availability

No data was generated for this review.

## References

[1] Bristow MR, Ginsburg R, Minobe W, Cubicciotti RS, Sageman WS, Lurie K, Billingham ME, Harrison DC, Stinson EB. Decreased catecholamine sensitivity and beta-adrenergic-receptor density in failing human hearts. N Engl J Med. 1982;307:205–211. doi: 10.1056/NEJM198207223070401 6283349

[2] Bristow MR, Ginsburg R, Umans V, Fowler M, Minobe W, Rasmussen R, Zera P, Menlove R, Shah P, Jamieson S, Beta 1- and beta 2-adrenergic-receptor subpopulations in nonfailing and failing human ventricular myocardium: coupling of both receptor subtypes to muscle contraction and selective beta 1-receptor down-regulation in heart failure. Circ Res. 1986;59:297–309. doi: 10.1161/01.res.59.3.297 2876788

[3] Ungerer M, Bohm M, Elce JS, Erdmann E, Lohse MJ. Altered expression of beta-adrenergic receptor kinase and beta 1-adrenergic receptors in the failing human heart. Circulation. 1993;87:454–463. doi: 10.1161/01.cir.87.2.454 8381058

[4] Bristow MR. beta-adrenergic receptor blockade in chronic heart failure. Circulation. 2000;101:558–569. doi: 10.1161/01.cir.101.5.558 10662755

[5] Iaccarino G, Barbato E, Cipolletta E, De Amicis V, Margulies KB, Leosco D, Trimarco B, Koch WJ. Elevated myocardial and lymphocyte GRK2 expression and activity in human heart failure. Eur Heart J. 2005;26:1752–1758. doi: 10.1093/eurheartj/ehi429 16055494

[6] Belmonte SL, Blaxall BC. G protein coupled receptor kinases as therapeutic targets in cardiovascular disease. Circ Res. 2011;109:309–319. doi: 10.1161/CIRCRESAHA.110.231233 21778435 PMC3146028

[7] Sato PY, Chuprun JK, Schwartz M, Koch WJ. The evolving impact of g protein-coupled receptor kinases in cardiac health and disease. Physiol Rev. 2015;95:377–404. doi: 10.1152/physrev.00015.2014 25834229 PMC4551214

[8] Aguero J, Almenar L, Monto F, Oliver E, Sanchez-Lazaro I, Vicente D, Martinez-Dolz L, D'Ocon P, Rueda J, Salvador A. Myocardial G protein receptor-coupled kinase expression correlates with functional parameters and clinical severity in advanced heart failure. J Card Fail. 2012;18:53–61. doi: 10.1016/j.cardfail.2011.10.008 22196842

[9] Penela P, Inserte J, Ramos P, Rodriguez-Sinovas A, Garcia-Dorado D, Mayor F Jr,. Degradation of GRK2 and AKT is an early and detrimental event in myocardial ischemia/reperfusion. EBioMedicine. 2019;48:605–618. doi: 10.1016/j.ebiom.2019.09.019 31594751 PMC6838402

[10] Woodall MC, Woodall BP, Gao E, Yuan A, Koch WJ. Cardiac Fibroblast GRK2 Deletion Enhances Contractility and Remodeling Following Ischemia/Reperfusion Injury. Circ Res. 2016;119:1116–1127. doi: 10.1161/CIRCRESAHA.116.309538 27601479 PMC5085864

[11] Chen M, Sato PY, Chuprun JK, Peroutka RJ, Otis NJ, Ibetti J, Pan S, Sheu SS, Gao E, Koch WJ. Prodeath signaling of G protein-coupled receptor kinase 2 in cardiac myocytes after ischemic stress occurs via extracellular signal-regulated kinase-dependent heat shock protein 90-mediated mitochondrial targeting. Circ Res. 2013;112:1121–1134. doi: 10.1161/CIRCRESAHA.112.300754 23467820 PMC3908784

[12] Sato PY, Chuprun JK, Grisanti LA, Woodall MC, Brown BR, Roy R, Traynham CJ, Ibetti J, Lucchese AM, Yuan A, Drosatos K, Tilley DG, Gao E, Koch WJ. Restricting mitochondrial GRK2 post-ischemia confers cardioprotection by reducing myocyte death and maintaining glucose oxidation. Sci Signal. 2018;11. doi: 10.1126/scisignal.aau0144 PMC6463290 30538174

[13] Rengo G, Galasso G, Femminella GD, Parisi V, Zincarelli C, Pagano G, De Lucia C, Cannavo A, Liccardo D, Marciano C, Vigorito C, Giallauria F, Ferrara N, Furgi G, Filardi PP, Koch WJ, Leosco D. Reduction of lymphocyte G protein-coupled receptor kinase-2 (GRK2) after exercise training predicts survival in patients with heart failure. Eur J Prev Cardiol. 2014;21:4–11. doi: 10.1177/2047487313491656 23689525

[14] Koch WJ, Rockman HA, Samama P, Hamilton RA, Bond RA, Milano CA, Lefkowitz RJ. Cardiac function in mice overexpressing the beta-adrenergic receptor kinase or a beta ARK inhibitor. Science. 1995;268:1350–1353. doi: 10.1126/science.7761854 7761854

[15] Kuai J, Han C, Wei W. Potential Regulatory Roles of GRK2 in Endothelial Cell Activity and Pathological Angiogenesis. Front Immunol. 2021;12:698424. doi: 10.3389/fimmu.2021.698424 34335610 PMC8320431

[16] Wu LD, Shi Y, Kong CH, Chen AQ, Kang Y, Kan JY, Jiang XM, Chu P, Wang DC, Lv YF, Qian ZY, Jiang ZH, Chen YW, Sun Y, Chang RR, Zhou WY, Gu Y, Zhang JX, Chen SL. The GRK2/AP-1 Signaling Axis Mediates Vascular Endothelial Dysfunction and Atherosclerosis Induced by Oscillatory Low Shear Stress. Adv Sci (Weinh). 2025;12:e01981. doi: 10.1002/advs.202501981 40492401 PMC12412516

[17] Yang XZ, Zhang WK, Zhu ZQ, Zhao W, Luo LL, Wang LP, Zhao YJ, Chang Y, Wei W. GRK2-mediated phosphorylation and de-succinylation of PKM2 reduce macrophage glycolysis in rheumatoid arthritis. Acta Pharmacol Sin. 2025;46:2693–2706. doi: 10.1038/s41401-025-01582-y 40425786 PMC12460887

[18] Zhang J, Zhang X, Lu M, Chang Y, Wang Q, Tu J, Wu H, Wang C, Hong Z, Xiong M, Song L, Wei W. GRK2 Mediates Macrophage Polarization by Regulating EP4-cAMP-pCREB Signaling in Ulcerative Colitis and the Therapeutic Effect of Paroxetine on Mice with DSS-Induced Colitis. Pharmaceuticals (Basel). 2023;16:664. doi: 10.3390/ph16050664 37242446 PMC10223763

[19] Glaser KM, Tarrant TK, Lammermann T. Combinatorial depletions of G-protein coupled receptor kinases in immune cells identify pleiotropic and cell type-specific functions. Front Immunol. 2022;13:1039803. doi: 10.3389/fimmu.2022.1039803 36451830 PMC9703078

[20] Travers JG, Schafer AE, Blaxall BC. GRK2 in Lymphocytes: Expanding the Arsenal of Heart Failure Prognostics. Circ Res. 2016;118:1049–1051. doi: 10.1161/CIRCRESAHA.116.308542 27034271 PMC4819241

[21] Bhutani V, Varzideh F, Wilson S, Kansakar U, Jankauskas SS, Santulli G. Doxorubicin-Induced Cardiotoxicity: A Comprehensive Update. J Cardiovasc Dev Dis. 2025;12. doi: 10.3390/jcdd12060207 PMC12193598 40558642

[22] Sheibani M, Azizi Y, Shayan M, Nezamoleslami S, Eslami F, Farjoo MH, Dehpour AR. Doxorubicin-Induced Cardiotoxicity: An Overview on Pre-clinical Therapeutic Approaches. Cardiovasc Toxicol. 2022;22:292–310. doi: 10.1007/s12012-022-09721-1 35061218

[23] Palaskas NL, Ali HJ, Koutroumpakis E, Ganatra S, Deswal A. Cardiovascular toxicity of immune therapies for cancer. BMJ. 2024;385:e075859. doi: 10.1136/bmj-2023-075859 38749554

[24] Lymperopoulos A, Rengo G, Funakoshi H, Eckhart AD, Koch WJ. Adrenal GRK2 upregulation mediates sympathetic overdrive in heart failure. Nat Med. 2007;13:315–323. doi: 10.1038/nm1553 17322894

[25] Lymperopoulos A, Rengo G, Gao E, Ebert SN, Dorn GW, 2nd, Koch WJ. Reduction of sympathetic activity via adrenal-targeted GRK2 gene deletion attenuates heart failure progression and improves cardiac function after myocardial infarction. J Biol Chem. 2010;285:16378–16386. doi: 10.1074/jbc.M109.077859 20351116 PMC2871505

[26] Rengo G, Leosco D, Zincarelli C, Marchese M, Corbi G, Liccardo D, Filippelli A, Ferrara N, Lisanti MP, Koch WJ, Lymperopoulos A. Adrenal GRK2 lowering is an underlying mechanism for the beneficial sympathetic effects of exercise training in heart failure. Am J Physiol Heart Circ Physiol. 2010;298:H2032–2038. doi: 10.1152/ajpheart.00702.2009 20304818

[27] Tachibana H, Naga Prasad SV, Lefkowitz RJ, Koch WJ, Rockman HA. Level of beta-adrenergic receptor kinase 1 inhibition determines degree of cardiac dysfunction after chronic pressure overload-induced heart failure. Circulation. 2005;111:591–597. doi: 10.1161/01.CIR.0000142291.70954.DF 15668342

[28] Eckhart AD, Koch WJ. Expression of a beta-adrenergic receptor kinase inhibitor reverses dysfunction in failing cardiomyocytes. Mol Ther. 2002;5:74–79. doi: 10.1006/mthe.2001.0508 11786048

[29] Rockman HA, Chien KR, Choi DJ, Iaccarino G, Hunter JJ, Ross J, Jr., Lefkowitz RJ, Koch WJ. Expression of a beta-adrenergic receptor kinase 1 inhibitor prevents the development of myocardial failure in gene-targeted mice. Proc Natl Acad Sci U S A. 1998;95:7000–7005. doi: 10.1073/pnas.95.12.7000 9618528 PMC22717

[30] Abd Alla J, Graemer M, Fu X, Quitterer U. Inhibition of G-protein-coupled Receptor Kinase 2 Prevents the Dysfunctional Cardiac Substrate Metabolism in Fatty Acid Synthase Transgenic Mice. J Biol Chem. 2016;291:2583–2600. doi: 10.1074/jbc.M115.702688 26670611 PMC4742729

[31] Roy R, Schumacher SM, Murphy HC, Grondolsky J, Rosales TO, Chuprun JK, Gao E, Zhao H, Berretta RM, Hobby ARH, Houser SR, Avramova LV, Tesmer JJG, Koch WJ. Therapeutic Efficacy of a Novel Pharmacologic GRK2 Inhibitor in Multiple Animal Models of Heart Failure. JACC Basic Transl Sci. 2025;10:202–217. doi: 10.1016/j.jacbts.2024.10.008 40131155 PMC11897459

[32] Schumacher SM, Gao E, Zhu W, Chen X, Chuprun JK, Feldman AM, Tesmer JJ, Koch WJ. Paroxetine-mediated GRK2 inhibition reverses cardiac dysfunction and remodeling after myocardial infarction. Sci Transl Med. 2015;7:277ra231. doi: 10.1126/scitranslmed.aaa0154 PMC4768806 25739765

[33] Raake PW, Schlegel P, Ksienzyk J, Reinkober J, Barthelmes J, Schinkel S, Pleger S, Mier W, Haberkorn U, Koch WJ, Katus HA, Most P, Muller OJ. AAV6.betaARKct cardiac gene therapy ameliorates cardiac function and normalizes the catecholaminergic axis in a clinically relevant large animal heart failure model. Eur Heart J. 2013;34:1437–1447. doi: 10.1093/eurheartj/ehr447 22261894 PMC3653122

[34] Raake PW, Vinge LE, Gao E, Boucher M, Rengo G, Chen X, DeGeorge BR Jr., Matkovich S, Houser SR, Most P, Eckhart AD, Dorn GW 2nd, Koch WJ. G protein-coupled receptor kinase 2 ablation in cardiac myocytes before or after myocardial infarction prevents heart failure. Circ Res. 2008;103:413–422. doi: 10.1161/CIRCRESAHA.107.168336 18635825 PMC2679955

[35] Fu X, Koller S, Abd Alla J, Quitterer U. Inhibition of G-protein-coupled receptor kinase 2 (GRK2) triggers the growth-promoting mitogen-activated protein kinase (MAPK) pathway. J Biol Chem. 2013;288:7738–7755. doi: 10.1074/jbc.M112.428078 23362259 PMC3597814

[36] Thal DM, Homan KT, Chen J, Wu EK, Hinkle PM, Huang ZM, Chuprun JK, Song J, Gao E, Cheung JY, Sklar LA, Koch WJ, Tesmer JJ. Paroxetine is a direct inhibitor of g protein-coupled receptor kinase 2 and increases myocardial contractility. Acs Chem Biol. 2012;7:1830–1839. doi: 10.1021/cb3003013 22882301 PMC3500392

[37] Homan KT, Wu E, Wilson MW, Singh P, Larsen SD, Tesmer JJ. Structural and functional analysis of g protein-coupled receptor kinase inhibition by paroxetine and a rationally designed analog. Mol Pharmacol. 2014;85:237–248. doi: 10.1124/mol.114.091488 24220010 PMC3913355

[38] Sato PY, Chuprun JK, Ibetti J, Cannavo A, Drosatos K, Elrod JW, Koch WJ. GRK2 compromises cardiomyocyte mitochondrial function by diminishing fatty acid-mediated oxygen consumption and increasing superoxide levels. J Mol Cell Cardiol. 2015;89:360–364. doi: 10.1016/j.yjmcc.2015.10.002 26506135 PMC4689631

[39] Casey LM, Pistner AR, Belmonte SL, Migdalovich D, Stolpnik O, Nwakanma FE, Vorobiof G, Dunaevsky O, Matavel A, Lopes CM, Smrcka AV, Blaxall BC. Small molecule disruption of G beta gamma signaling inhibits the progression of heart failure. Circ Res. 2010;107:532–539. doi: 10.1161/CIRCRESAHA.110.217075 20576935 PMC2924955

[40] Lopaschuk GD, Karwi QG, Tian R, Wende AR, Abel ED. Cardiac Energy Metabolism in Heart Failure. Circ Res. 2021;128:1487–1513. doi: 10.1161/CIRCRESAHA.121.318241 33983836 PMC8136750

[41] Ciccarelli M, Chuprun JK, Rengo G, Gao E, Wei Z, Peroutka RJ, Gold JI, Gumpert A, Chen M, Otis NJ, Dorn GW, 2nd, Trimarco B, Iaccarino G, Koch WJ. G protein-coupled receptor kinase 2 activity impairs cardiac glucose uptake and promotes insulin resistance after myocardial ischemia. Circulation. 2011;123:1953–1962. doi: 10.1161/CIRCULATIONAHA.110.988642 21518983 PMC3113597

[42] Snyder J, Jiang CS, Choi RH, Morgan T, Roman J, Underwood L, Lucchese AM, Montgomery S, Grisanti LA, Doliba N, Holland WL, Sato PY. Cardioprotective effect of genetic ablation of the G-protein-coupled receptor kinase GRK2 in adult pancreatic beta-cells during high-fat diet. J Biol Chem. 2025;301:108388. doi: 10.1016/j.jbc.2025.108388 40054692 PMC12018985

[43] Ciccarelli M, Sorriento D, Fiordelisi A, Gambardella J, Franco A, Del Giudice C, Sala M, Monti MG, Bertamino A, Campiglia P, Oliveti M, Poggio P, Trinchese G, Cavaliere G, Cipolletta E, Mollica MP, Bonaduce D, Trimarco B, Iaccarino G. Pharmacological inhibition of GRK2 improves cardiac metabolism and function in experimental heart failure. ESC Heart Fail. 2020;7:1571–1584. doi: 10.1002/ehf2.12706 32352228 PMC7373898

[44] Motso A, Pelcman B, Kalinovich A, Kahlous NA, Bokhari MH, Dehvari N, Halleskog C, Waara E, de Jong J, Cheesman E, Kallenberg C, Yakala GK, Murad P, Wetterdal E, Andersson P, van Beek S, Sandstrom A, Alleluia DN, Talamonti E, Youhanna S, Sabatier P, Koenig C, Willems S, Kemas AM, Hutchinson DS, Ham S, Gratz L, Voss J, Marchan-Alvarez JG, Priede M, Jaunsleine K, Spura J, Kovada V, Supe L, Stoddart LA, Holliday ND, Newton PT, Pillon NJ, Schulte G, Summers RJ, Mutule I, Suna E, Olsen JV, Molenaar P, Carlsson J, Lauschke VM, Wright SC, Bengtsson T. GRK-biased adrenergic agonists for the treatment of type 2 diabetes and obesity. Cell. 2025;188:5142–5156 e5123. doi: 10.1016/j.cell.2025.05.042 40555230

[45] Lucas E, Jurado-Pueyo M, Fortuno MA, Fernandez-Veledo S, Vila-Bedmar R, Jimenez-Borreguero LJ, Lazcano JJ, Gao E, Gomez-Ambrosi J, Fruhbeck G, Koch WJ, Diez J, Mayor F Jr., Murga C. Downregulation of G protein-coupled receptor kinase 2 levels enhances cardiac insulin sensitivity and switches on cardioprotective gene expression patterns. Biochim Biophys Acta. 2014;1842:2448–2456. doi: 10.1016/j.bbadis.2014.09.004 25239306

[46] Arcones AC, Vila-Bedmar R, Mirasierra M, Cruces-Sande M, Vallejo M, Jones B, Tomas A, Mayor F Jr., Murga C. GRK2 regulates GLP-1R-mediated early phase insulin secretion in vivo. BMC Biol. 2021;19:40. doi: 10.1186/s12915-021-00966-w 33658023 PMC7931601

[47] Cannavo A, Liccardo D, Lymperopoulos A, Santangelo M, Femminella GD, Leosco D, Cittadini A, Ferrara N, Paolocci N, Koch WJ, Rengo G. GRK2 Regulates alpha(2)-Adrenergic Receptor-Dependent Catecholamine Release in Human Adrenal Chromaffin Cells. J Am Coll Cardiol. 2017;69:1515–1517. doi: 10.1016/j.jacc.2017.01.016 28302297 PMC6645914

[48] Luo J, Benovic JL. G protein-coupled receptor kinase interaction with Hsp90 mediates kinase maturation. J Biol Chem. 2003;278:50908–50914. doi: 10.1074/jbc.M307637200 14557268

[49] Fan Q, Chen M, Zuo L, Shang X, Huang MZ, Ciccarelli M, Raake P, Brinks H, Chuprun KJ, Dorn GW 2nd, Koch WJ, Gao E. Myocardial Ablation of G Protein-Coupled Receptor Kinase 2 (GRK2) Decreases Ischemia/Reperfusion Injury through an Anti-Intrinsic Apoptotic Pathway. PLoS One. 2013;8:e66234. doi: 10.1371/journal.pone.0066234 23805205 PMC3689757

[50] Brinks H, Boucher M, Gao E, Chuprun JK, Pesant S, Raake PW, Huang ZM, Wang X, Qiu G, Gumpert A, Harris DM, Eckhart AD, Most P, Koch WJ. Level of G protein-coupled receptor kinase-2 determines myocardial ischemia/reperfusion injury via pro- and anti-apoptotic mechanisms. Circ Res. 2010;107:1140–1149. doi: 10.1161/CIRCRESAHA.110.221010 20814022 PMC2966514

[51] Nandagopal K, Dawson TM, Dawson VL. Critical role for nitric oxide signaling in cardiac and neuronal ischemic preconditioning and tolerance. J Pharmacol Exp Ther. 2001;297:474–478. 11303032

[52] Schulz R, Kelm M, Heusch G. Nitric oxide in myocardial ischemia/reperfusion injury. Cardiovasc Res. 2004;61:402–413. doi: 10.1016/j.cardiores.2003.09.019 14962472

[53] Gould N, Doulias PT, Tenopoulou M, Raju K, Ischiropoulos H. Regulation of protein function and signaling by reversible cysteine S-nitrosylation. J Biol Chem. 2013;288:26473–26479. doi: 10.1074/jbc.R113.460261 23861393 PMC3772194

[54] Huang ZM, Gao E, Fonseca FV, Hayashi H, Shang X, Hoffman NE, Chuprun JK, Tian X, Tilley DG, Madesh M, Lefer DJ, Stamler JS, Koch WJ. Convergence of G protein-coupled receptor and S-nitrosylation signaling determines the outcome to cardiac ischemic injury. Sci Signal. 2013;6:ra95. doi: 10.1126/scisignal.2004225 24170934 PMC3969021

[55] Whalen EJ, Foster MW, Matsumoto A, Ozawa K, Violin JD, Que LG, Nelson CD, Benhar M, Keys JR, Rockman HA, Koch WJ, Daaka Y, Lefkowitz RJ, Stamler JS. Regulation of beta-adrenergic receptor signaling by S-nitrosylation of G-protein-coupled receptor kinase 2. Cell. 2007;129:511–522. doi: 10.1016/j.cell.2007.02.046 17482545

[56] Lieu M, Traynham CJ, de Lucia C, Pfleger J, Piedepalumbo M, Roy R, Petovic J, Landesberg G, Forrester SJ, Hoffman M, Grisanti LA, Yuan A, Gao E, Drosatos K, Eguchi S, Scalia R, Tilley DG, Koch WJ. Loss of dynamic regulation of G protein-coupled receptor kinase 2 by nitric oxide leads to cardiovascular dysfunction with aging. Am J Physiol Heart Circ Physiol. 2020;318:H1162–H1175. doi: 10.1152/ajpheart.00094.2020 32216616 PMC7346533

[57] Krasel C, Dammeier S, Winstel R, Brockmann J, Mischak H, Lohse MJ. Phosphorylation of GRK2 by protein kinase C abolishes its inhibition by calmodulin. J Biol Chem. 2001;276:1911–1915. doi: 10.1074/jbc.M008773200 11042191

[58] Ciccarelli M, Sorriento D, Franco A, Fusco A, Del Giudice C, Annunziata R, Cipolletta E, Monti MG, Dorn GW, 2nd, Trimarco B, Iaccarino G. Endothelial G protein-coupled receptor kinase 2 regulates vascular homeostasis through the control of free radical oxygen species. Arterioscler Thromb Vasc Biol. 2013;33:2415–2424. doi: 10.1161/ATVBAHA.113.302262 23950144 PMC4262246

[59] Liu S, Premont RT, Kontos CD, Zhu S, Rockey DC. A crucial role for GRK2 in regulation of endothelial cell nitric oxide synthase function in portal hypertension. Nat Med. 2005;11:952–958. doi: 10.1038/nm1289 16142243

[60] Dick SA, Macklin JA, Nejat S, Momen A, Clemente-Casares X, Althagafi MG, Chen J, Kantores C, Hosseinzadeh S, Aronoff L, Wong A, Zaman R, Barbu I, Besla R, Lavine KJ, Razani B, Ginhoux F, Husain M, Cybulsky MI, Robbins CS, Epelman S. Self-renewing resident cardiac macrophages limit adverse remodeling following myocardial infarction. Nat Immunol. 2019;20:29–39. doi: 10.1038/s41590-018-0272-2 30538339 PMC6565365

[61] Li W, Hsiao HM, Higashikubo R, Saunders BT, Bharat A, Goldstein DR, Krupnick AS, Gelman AE, Lavine KJ, Kreisel D. Heart-resident CCR2(+) macrophages promote neutrophil extravasation through TLR9/MyD88/CXCL5 signaling. Jci Insight. 2016;1. doi: 10.1172/jci.insight.87315 PMC4985028 27536731

[62] Nahrendorf M, Swirski FK, Aikawa E, Stangenberg L, Wurdinger T, Figueiredo JL, Libby P, Weissleder R, Pittet MJ. The healing myocardium sequentially mobilizes two monocyte subsets with divergent and complementary functions. J Exp Med. 2007;204:3037–3047. doi: 10.1084/jem.20070885 18025128 PMC2118517

[63] Stevenson NL, Martin-Martin B, Freeman J, Kriston-Vizi J, Ketteler R, Cutler DF. G protein-coupled receptor kinase 2 moderates recruitment of THP-1 cells to the endothelium by limiting histamine-invoked Weibel-Palade body exocytosis. J Thromb Haemost. 2014;12:261–272. doi: 10.1111/jth.12470 PMC4238739 24735118

[64] Cassarino MC, Colado A, Martinez VS, Martines C, Bonato A, Bertini M, Pavlovksy M, Custidiano R, Bezares FR, Morande PE, Vermeulen M, Gamberale R, Giordano M, Efremov DG, Borge M. G-protein coupled receptor kinase-2 regulates the migration of chronic lymphocytic leukaemia cells to sphingosine-1 phosphate in vitro and their trafficking in vivo. Sci Rep. 2025;15:6530. doi: 10.1038/s41598-025-91536-5 39988601 PMC11847938

[65] Cheng J, Lucas PC, McAllister-Lucas LM. Canonical and Non-Canonical Roles of GRK2 in Lymphocytes. Cells. 2021;10. doi: 10.3390/cells10020307 PMC7913175 33546162

[66] Lavine KJ, Epelman S, Uchida K, Weber KJ, Nichols CG, Schilling JD, Ornitz DM, Randolph GJ, Mann DL. Distinct macrophage lineages contribute to disparate patterns of cardiac recovery and remodeling in the neonatal and adult heart. Proc Natl Acad Sci U S A. 2014;111:16029–16034. doi: 10.1073/pnas.1406508111 25349429 PMC4234568

[67] Kadyrov FF, Koenig AL, Amrute JM, Dun H, Li W, Weinheimer CJ, Nigro JM, Kovacs A, Bredemeyer AL, Yang S, Das S, Penna VR, Parvathaneni A, Lai L, Hartmann N, Kopecky BJ, Kreisel D, Lavine KJ. Hypoxia sensing in resident cardiac macrophages regulates monocyte fate specification following ischemic heart injury. Nat Cardiovasc Res. 2024;3:1337–1355. doi: 10.1038/s44161-024-00553-6 39433910 PMC12696718

[68] Liu Y, Xu R, Gu H, Zhang E, Qu J, Cao W, Huang X, Yan H, He J, Cai Z. Metabolic reprogramming in macrophage responses. Biomark Res. 2021;9:1. doi: 10.1186/s40364-020-00251-y 33407885 PMC7786975

[69] Van den Bossche J, O'Neill LA, Menon D. Macrophage Immunometabolism: Where Are We (Going)? Trends Immunol. 2017;38:395–406. doi: 10.1016/j.it.2017.03.001 28396078

[70] Yan X, Anzai A, Katsumata Y, Matsuhashi T, Ito K, Endo J, Yamamoto T, Takeshima A, Shinmura K, Shen W, Fukuda K, Sano M. Temporal dynamics of cardiac immune cell accumulation following acute myocardial infarction. J Mol Cell Cardiol. 2013;62:24–35. doi: 10.1016/j.yjmcc.2013.04.023 23644221

[71] de Winter N, Ji J, Sintou A, Forte E, Lee M, Noseda M, Li A, Koenig AL, Lavine KJ, Hayat S, Rosenthal N, Emanueli C, Srivastava PK, Sattler S. Persistent transcriptional changes in cardiac adaptive immune cells following myocardial infarction: New evidence from the re-analysis of publicly available single cell and nuclei RNA-sequencing data sets. J Mol Cell Cardiol. 2024;192:48–64. doi: 10.1016/j.yjmcc.2024.04.016 38734060

[72] Dinkel BA, Kremer KN, Rollins MR, Medlyn MJ, Hedin KE. GRK2 mediates TCR-induced transactivation of CXCR4 and TCR-CXCR4 complex formation that drives PI3Kgamma/PREX1 signaling and T cell cytokine secretion. J Biol Chem. 2018;293:14022–14039. doi: 10.1074/jbc.RA118.003097 30018141 PMC6130939

[73] Arnon TI, Xu Y, Lo C, Pham T, An J, Coughlin S, Dorn GW, Cyster JG. GRK2-dependent S1PR1 desensitization is required for lymphocytes to overcome their attraction to blood. Science. 2011;333:1898–1903. doi: 10.1126/science.1208248 21960637 PMC3267326

[74] Herrmann J, Lenihan D, Armenian S, Barac A, Blaes A, Cardinale D, Carver J, Dent S, Ky B, Lyon AR, Lopez-Fernandez T, Fradley MG, Ganatra S, Curigliano G, Mitchell JD, Minotti G, Lang NN, Liu JE, Neilan TG, Nohria A, O'Quinn R, Pusic I, Porter C, Reynolds KL, Ruddy KJ, Thavendiranathan P, Valent P. Defining cardiovascular toxicities of cancer therapies: an International Cardio-Oncology Society (IC-OS) consensus statement. Eur Heart J. 2022;43:280–299. doi: 10.1093/eurheartj/ehab674 34904661 PMC8803367

[75] Lyon AR, Lopez-Fernandez T, Couch LS, Asteggiano R, Aznar MC, Bergler-Klein J, Boriani G, Cardinale D, Cordoba R, Cosyns B, Cutter DJ, de Azambuja E, de Boer RA, Dent SF, Farmakis D, Gevaert SA, Gorog DA, Herrmann J, Lenihan D, Moslehi J, Moura B, Salinger SS, Stephens R, Suter TM, Szmit S, Tamargo J, Thavendiranathan P, Tocchetti CG, van der Meer P, van der Pal HJH, Group ESCSD. 2022 ESC Guidelines on cardio-oncology developed in collaboration with the European Hematology Association (EHA), the European Society for Therapeutic Radiology and Oncology (ESTRO) and the International Cardio-Oncology Society (IC-OS). Eur Heart J. 2022;43:4229–4361. doi: 10.1093/eurheartj/ehac244 36017568

[76] Babuin L, Jaffe AS. Troponin: the biomarker of choice for the detection of cardiac injury. CMAJ. 2005;173:1191–1202. doi: 10.1503/cmaj/051291 16275971 PMC1277047

[77] Cardinale D, Colombo A, Sandri MT, Lamantia G, Colombo N, Civelli M, Martinelli G, Veglia F, Fiorentini C, Cipolla CM. Prevention of high-dose chemotherapy-induced cardiotoxicity in high-risk patients by angiotensin-converting enzyme inhibition. Circulation. 2006;114:2474–2481. doi: 10.1161/CIRCULATIONAHA.106.635144 17101852

[78] Cardinale D, Ciceri F, Latini R, Franzosi MG, Sandri MT, Civelli M, Cucchi G, Menatti E, Mangiavacchi M, Cavina R, Barbieri E, Gori S, Colombo A, Curigliano G, Salvatici M, Rizzo A, Ghisoni F, Bianchi A, Falci C, Aquilina M, Rocca A, Monopoli A, Milandri C, Rossetti G, Bregni M, Sicuro M, Malossi A, Nassiacos D, Verusio C, Giordano M, Staszewsky L, Barlera S, Nicolis EB, Magnoli M, Masson S, Cipolla CM, Investigators I-OS. Anthracycline-induced cardiotoxicity: A multicenter randomised trial comparing two strategies for guiding prevention with enalapril: The International CardioOncology Society-one trial. Eur J Cancer. 2018;94:126–137. doi: 10.1016/j.ejca.2018.02.012 29567630

[79] Henriksen PA, Hall P, Oikonomidou O, MacPherson IR, Maclean M, Lewis S, McVicars H, Broom A, Scott F, McKay P, Borley A, Rowntree C, Lord S, Collins G, Radford J, Guppy A, Payne JR, Newby DE, Mills NL, Lang NN. Rationale and Design of the Cardiac CARE Trial: A Randomized Trial of Troponin-Guided Neurohormonal Blockade for the Prevention of Anthracycline Cardiotoxicity. Circ Heart Fail. 2022;15:e009445. doi: 10.1161/CIRCHEARTFAILURE.121.009445 35766037

[80] Gulati G, Heck SL, Ree AH, Hoffmann P, Schulz-Menger J, Fagerland MW, Gravdehaug B, von Knobelsdorff-Brenkenhoff F, Bratland A, Storas TH, Hagve TA, Rosjo H, Steine K, Geisler J, Omland T. Prevention of cardiac dysfunction during adjuvant breast cancer therapy (PRADA): a 2 × 2 factorial, randomized, placebo-controlled, double-blind clinical trial of candesartan and metoprolol. Eur Heart J. 2016;37:1671–1680. doi: 10.1093/eurheartj/ehw022 26903532 PMC4887703

[81] Avila MS, Ayub-Ferreira SM, de Barros Wanderley MR Jr, das Dores Cruz F, Goncalves Brandao SM, Rigaud VOC, Higuchi-Dos-Santos MH, Hajjar LA, Kalil Filho R, Hoff PM, Sahade M, Ferrari MSM, de Paula Costa RL, Mano MS, Bittencourt Viana Cruz CB, Abduch MC, Lofrano Alves MS, Guimaraes GV, Issa VS, Bittencourt MS, Bocchi EA. Carvedilol for Prevention of Chemotherapy-Related Cardiotoxicity: The CECCY Trial. J Am Coll Cardiol. 2018;71:2281–2290. doi: 10.1016/j.jacc.2018.02.049 29540327

[82] Swain SM, Whaley FS, Gerber MC, Weisberg S, York M, Spicer D, Jones SE, Wadler S, Desai A, Vogel C, Speyer J, Mittelman A, Reddy S, Pendergrass K, Velez-Garcia E, Ewer MS, Bianchine JR, Gams RA. Cardioprotection with dexrazoxane for doxorubicin-containing therapy in advanced breast cancer. J Clin Oncol. 1997;15:1318–1332. doi: 10.1200/JCO.1997.15.4.1318 9193323

[83] Marty M, Espie M, Llombart A, Monnier A, Rapoport BL, Stahalova V, Dexrazoxane Study G. Multicenter randomized phase III study of the cardioprotective effect of dexrazoxane (Cardioxane) in advanced/metastatic breast cancer patients treated with anthracycline-based chemotherapy. Ann Oncol. 2006;17:614–622. doi: 10.1093/annonc/mdj134 16423847

[84] de Baat EC, Mulder RL, Armenian S, Feijen EA, Grotenhuis H, Hudson MM, Mavinkurve-Groothuis AM, Kremer LC, van Dalen EC. Dexrazoxane for preventing or reducing cardiotoxicity in adults and children with cancer receiving anthracyclines. Cochrane Database Syst Rev. 2022;9:CD014638. doi: 10.1002/14651858.CD014638.pub2 36162822 PMC9512638

[85] Macedo AVS, Hajjar LA, Lyon AR, Nascimento BR, Putzu A, Rossi L, Costa RB, Landoni G, Nogueira-Rodrigues A, Ribeiro ALP. Efficacy of Dexrazoxane in Preventing Anthracycline Cardiotoxicity in Breast Cancer. JACC CardioOncol. 2019;1:68–79. doi: 10.1016/j.jaccao.2019.08.003 34396164 PMC8352186

[86] Junjing Z, Yan Z, Baolu Z. Scavenging effects of dexrazoxane on free radicals. J Clin Biochem Nutr. 2010;47:238–245. doi: 10.3164/jcbn.10-64 21103033 PMC2966934

[87] Nielsen DL, Juhl CB, Nielsen OH, Chen IM, Herrmann J. Immune Checkpoint Inhibitor-Induced Cardiotoxicity: A Systematic Review and Meta-Analysis. JAMA Oncol. 2024;10:1390–1399. doi: 10.1001/jamaoncol.2024.3065 39172480 PMC11342217

[88] Bencivenga L, Palaia ME, Sepe I, Gambino G, Komici K, Cannavo A, Femminella GD, Rengo G. Why Do We Not Assess Sympathetic Nervous System Activity in Heart Failure Management: Might GRK2 Serve as a New Biomarker? Cells. 2021;10:457. doi: 10.3390/cells10020457 33669936 PMC7924864

[89] Johnson DB, Balko JM, Compton ML, Chalkias S, Gorham J, Xu Y, Hicks M, Puzanov I, Alexander MR, Bloomer TL, Becker JR, Slosky DA, Phillips EJ, Pilkinton MA, Craig-Owens L, Kola N, Plautz G, Reshef DS, Deutsch JS, Deering RP, Olenchock BA, Lichtman AH, Roden DM, Seidman CE, Koralnik IJ, Seidman JG, Hoffman RD, Taube JM, Diaz LA Jr., Anders RA, Sosman JA, Moslehi JJ. Fulminant Myocarditis with Combination Immune Checkpoint Blockade. N Engl J Med. 2016;375:1749–1755. doi: 10.1056/NEJMoa1609214 27806233 PMC5247797

[90] Mahmood SS, Fradley MG, Cohen JV, Nohria A, Reynolds KL, Heinzerling LM, Sullivan RJ, Damrongwatanasuk R, Chen CL, Gupta D, Kirchberger MC, Awadalla M, Hassan MZO, Moslehi JJ, Shah SP, Ganatra S, Thavendiranathan P, Lawrence DP, Groarke JD, Neilan TG. Myocarditis in Patients Treated With Immune Checkpoint Inhibitors. J Am Coll Cardiol. 2018;71:1755–1764. doi: 10.1016/j.jacc.2018.02.037 29567210 PMC6196725

[91] Fenioux C, Abbar B, Boussouar S, Bretagne M, Power JR, Moslehi JJ, Gougis P, Amelin D, Dechartres A, Lehmann LH, Courand PY, Cautela J, Alexandre J, Procureur A, Rozes A, Leonard-Louis S, Qin J, International ICIMR, Cheynier R, Charmeteau-De Muylder B, Redheuil A, Tubach F, Cadranel J, Milon A, Ederhy S, Similowski T, Johnson DB, Pizzo I, Catalan T, Benveniste O, Hayek SS, Allenbach Y, Rosenzwajg M, Dolladille C, Salem JE. Thymus alterations and susceptibility to immune checkpoint inhibitor myocarditis. Nat Med. 2023;29:3100–3110. doi: 10.1038/s41591-023-02591-2 37884625

[92] Palaskas NL, Segura A, Lelenwa L, Siddiqui BA, Subudhi SK, Lopez-Mattei J, Durand JB, Deswal A, Zhao B, Maximilian Buja L, Iliescu C. Immune checkpoint inhibitor myocarditis: elucidating the spectrum of disease through endomyocardial biopsy. Eur J Heart Fail. 2021;23:1725–1735. doi: 10.1002/ejhf.2265 34114291

[93] Delombaerde D, Vervloet D, Franssen C, Croes L, Gremonprez F, Prenen H, Peeters M, Vulsteke C. Clinical implications of isolated troponinemia following immune checkpoint inhibitor therapy. ESMO Open. 2021;6:100216. doi: 10.1016/j.esmoop.2021.100216 34271309 PMC8287144

[94] Munir AZ, Gutierrez A, Qin J, Lichtman AH, Moslehi JJ. Immune-checkpoint inhibitor-mediated myocarditis: CTLA4, PD1 and LAG3 in the heart. Nat Rev Cancer. 2024;24:540–553. doi: 10.1038/s41568-024-00715-5 38982146

[95] Ma P, Liu J, Qin J, Lai L, Heo GS, Luehmann H, Sultan D, Bredemeyer A, Bajapa G, Feng G, Jimenez J, He R, Parks A, Amrute J, Villanueva A, Liu Y, Lin CY, Mack M, Amancherla K, Moslehi J, Lavine KJ. Expansion of Pathogenic Cardiac Macrophages in Immune Checkpoint Inhibitor Myocarditis. Circulation. 2024;149:48–66. doi: 10.1161/CIRCULATIONAHA.122.062551 37746718 PMC11323830

[96] Huang YV, Sun Y, Chou H, Wagner N, Vitale MR, Bayer AL, Xu B, Lee D, Lin Z, Branche C, Waliany S, Neal JW, Wakelee HA, Witteles RM, Nguyen PK, Graves EE, Berry GJ, Alcaide P, Wu SM, Zhu H. Novel Therapeutic Approach Targeting CXCR3 to Treat Immunotherapy Myocarditis. Circ Res. 2025;136:473–490. doi: 10.1161/CIRCRESAHA.124.325652 39931812 PMC11867805

[97] Gardner J, Eiger DS, Hicks C, Choi I, Pham U, Chundi A, Namjoshi O, Rajagopal S. GPCR kinases differentially modulate biased signaling downstream of CXCR3 depending on their subcellular localization. Sci Signal. 2024;17:eadd9139. doi: 10.1126/scisignal.add9139 38349966 PMC10927030

[98] Hachem AM, Desai A, Beinart N, Ostos-Mendoza KC, Rodriguez ASL, de Leon Derby RD, Ebrahimi S, Palaskas NL. Updates in Diagnosis and Treatment of Immune Checkpoint Inhibitor Myocarditis. Curr Cardiol Rep. 2025;27:78. doi: 10.1007/s11886-025-02232-9 40178703

[99] Salem JE, Allenbach Y, Vozy A, Brechot N, Johnson DB, Moslehi JJ, Kerneis M. Abatacept for Severe Immune Checkpoint Inhibitor-Associated Myocarditis. N Engl J Med. 2019;380:2377–2379. doi: 10.1056/NEJMc1901677 31189043

[100] Zhang L, Zlotoff DA, Awadalla M, Mahmood SS, Nohria A, Hassan MZO, Thuny F, Zubiri L, Chen CL, Sullivan RJ, Alvi RM, Rokicki A, Murphy SP, Jones-O'Connor M, Heinzerling LM, Barac A, Forrestal BJ, Yang EH, Gupta D, Kirchberger MC, Shah SP, Rizvi MA, Sahni G, Mandawat A, Mahmoudi M, Ganatra S, Ederhy S, Zatarain-Nicolas E, Groarke JD, Tocchetti CG, Lyon AR, Thavendiranathan P, Cohen JV, Reynolds KL, Fradley MG, Neilan TG. Major Adverse Cardiovascular Events and the Timing and Dose of Corticosteroids in Immune Checkpoint Inhibitor-Associated Myocarditis. Circulation. 2020;141:2031–2034. doi: 10.1161/CIRCULATIONAHA.119.044703 32539614 PMC7301778

[101] Waldschmidt HV, Homan KT, Cato MC, Cruz-Rodriguez O, Cannavo A, Wilson MW, Song J, Cheung JY, Koch WJ, Tesmer JJ, Larsen SD. Structure-Based Design of Highly Selective and Potent G Protein-Coupled Receptor Kinase 2 Inhibitors Based on Paroxetine. J Med Chem. 2017;60:3052–3069. doi: 10.1021/acs.jmedchem.7b00112 28323425 PMC5641445

[102] Lai S, Fu X, Yang S, Zhang S, Lin Q, Zhang M, Chen H. G protein-coupled receptor kinase-2: A potential biomarker for early diabetic cardiomyopathy. J Diabetes. 2020;12:247–258. doi: 10.1111/1753-0407.12991 31680450 PMC7064927

